# Dental Depot

**Published:** 1862-04

**Authors:** 


### DENTAL DEPOT.

TOGETHER with other departments of business, we observe our dental depots are preparing to answer the increased demands that the improvement in times is certain to produce. And fully equal to the emergency is the establishment of our old friend, Dr. J. M. Brown. He is in receipt of a large supply of new stock, consisting of everything needed by the dentist, which he proposes to furnish to the profession at greatly reduced prices on former rates.

It is wholly useless for us to say anything in commendation of this pioneer establishment, for it is known to every body, at least in the West. T.

